# A Moralisation on Games

**Published:** 1919-01-11

**Authors:** 


### A MORALISATION ON GAMES.

(FROM A CORRESPONDENT.)

NEARLY all mankind's active games are played with a ball. Cricket, football, golf, polo, croquet, billiards, and others of all degrees and complexity, are instanced. Most other active games depend upon one chasing another with more or less organisation of the pursuit. Of this sort are the boyish games of chevy, cross-touch, and prisoner's base. These are curious facts and subjects for reflection, because of universality. Games seem to be an evolution of the inborn desire for play exhibited by young creatures of the higher species, and perhaps, though less obviously, by creatures low in the scale of species. Puppies, kittens, colts, calves, lambs, and kids all play, and their play has the features of pursuit and flight. In addition, in it is often mimic combat of which human sports may be an evolution, as, for instance, wrestling matches and boxing bouts. It is easy to imagine gradual elaboration of primitive play up to highly-organised games coming with increasing intelligence. Roundness of substances leads to easy displacement and rolling so simulating live motion and so attracting young and curious animals to pursuit. Thereafter comes comprehension of possibilities of fun and the purposeful setting in motion of rounded objects. With leaves and pebbles and bits of wood cats, dogs, and fox cubs will play delightfully and will set them in motion again and again; will throw them by movements of the head, and carrying pieces of cloth or the wing of a bird, will fly from a companion, who pursues and snatches away and flees in turn in a way suggestive of a three-quarter back at Rugby football.

Some birds are given to play, notably ravens, which delight to disport themselves in the wind. They will fly into ravines of mountain sides up which strong gusts are blowing on stormy days, and there will yield themselves to the blast to be carried up by it till far beyond the strong draught, and from the altitude reached will return to get carried up again and again, much as surf-bathers use the force of shoreward rolling waves. Observations such as these indicate man's kinship to his less intelligent fellow-creatures, and are revelations of the marvellous and beautiful uniformity of the results of the Cause of the universe. Flies, midges, butterflies, and some other insects often seem to play, but with the highly communal organisations of life exhibited by bees, ants, and wasps life seems to have become too serious and intent for playful relaxation. This also may be a revelation of the universe, a warning to mankind that it may be that a too-highly organised civilisation, though not incompatible with the content and quiet happiness that comes of performance of duty and function, may deprive life of joy and play, variety and laughter; a loss great for any gain.

